# Crystallization and Stereocomplexation of PLA-*mb*-PBS Multi-Block Copolymers

**DOI:** 10.3390/polym10010008

**Published:** 2017-12-22

**Authors:** Rosa M. D’Ambrosio, Rose Mary Michell, Rosica Mincheva, Rebeca Hernández, Carmen Mijangos, Philippe Dubois, Alejandro J. Müller

**Affiliations:** 1 Grupo de Polímeros USB, Departamento de Ciencia de los Materiales, Universidad Simón Bolívar, Apartado 89000, 1080-A Caracas, Venezuela; rdambrosio@usb.ve (R.M.D.); rmichell@usb.ve (R.M.M.); 2 Laboratory of Polymeric and Composite Materials, Center of Innovation and Research in Materials & Polymers (CIRMAP), University of Mons-Hainaut, Place du Parc 20, B-7000 Mons, Belgium; rosica.mincheva@umons.ac.be (R.M.); philippe.dubois@umons.ac.be (P.D.); 3 Instituto de Ciencia y Tecnología de Polímeros, CSIC, c/Juan de la Cierva 3, 28006 Madrid, Spain; rhernandez@ictp.csic.es (R.H.); cmijangos@ictp.csic.es (C.M.); 4 Polymat and Polymer Science and Technology Department, Faculty of Chemistry, University of the Basque Country UPV/EHU, Paseo Manuel de Lardizabal 3, 20018 Donostia-San Sebastián, Spain; 5 Ikerbasque, Basque Foundation for Science, 48013 Bilbao, Spain

**Keywords:** poly(lactic acid) (PLA), crystallization kinetics, stereocomplexes, crystallization in multi-block copolymers, polybutylene succinate (PBS)

## Abstract

The crystallization and morphology of PLA-*mb*-PBS copolymers and their corresponding stereocomplexes were studied. The effect of flexible blocks (i.e., polybutylene succinate, PBS) on the crystallization of the copolymers and stereocomplex formation were investigated using polarized light optical microscopy (PLOM), differential scanning calorimetry (DSC), infrared spectroscopy (FTIR), and carbon-13 nuclear magnetic resonance spectroscopy (^13^C-NMR). The PLA and PBS multiple blocks were miscible in the melt and in the glassy state. When the PLA-*mb*-PBS copolymers are cooled from the melt, the PLA component crystallizes first creating superstructures, such as spherulites or axialites, which constitute a template within which the PBS component has to crystallize when the sample is further cooled down. The Avrami theory was able to fit the overall crystallization kinetics of both semi-crystalline components, and the *n* values for both blocks in all the samples had a correspondence with the superstructural morphology observed by PLOM. Solution mixtures of PLLA-*mb*-PBS and PLDA-*mb*-PBS copolymers were prepared, as well as copolymer/homopolymer blends with the aim to study the stereocomplexation of PLLA and PDLA chain segments. A lower amount of stereocomplex formation was observed in copolymer mixtures as compared to neat L_100_/D_100_ stereocomplexes. The results show that PBS chain segments perturb the formation of stereocomplexes and this perturbation increases with the amount of PBS in the samples. However, when relatively low amounts of PBS in the copolymer blends are present, the rate of stereocomplex formation is enhanced. This effect dissappears when higher amounts of PBS are present. The stereocomplexation was confirmed by FTIR and solid state ^13^C-NMR analyses.

## 1. Introduction

Poly(lactic acid) (PLA) is a biodegradable and biocompatible polymer that can be obtained from renewable resources and represents an interesting alternative for replacing petroleum-based polymers [1]. PLA is usually obtained in the amorphous state after being processed, in view of its slow crystallization rate in comparison to the cooling rates employed during processing. The resulting physical properties limit PLA practical applications, as the amorphous material has a low distortion temperature (related to its low *T_g_* value of around 55 °C) and is fragile at room temperature.

PLA has three different stereoisomers, the poly(l-lactide) (PLLA), poly(d-lactide) (PDLA), and the poly(l,d-lactide). The first two can crystallize, while the last one is always amorphous as it has no chain regularity for crystallization. Another interesting fact about PLA is the possibility to form stereocomplexes from the equimolecular mixture of PLLA and PDLA. The stereocomplex is a crystal formed by the helical configuration of the PLLA and PDLA chains assembled together in the same unit cells (i.e., co-crystallization) and has a higher stability than the PLA homocrystals, with melting temperatures that are approximately 50 °C higher than those of PLA homocrystals [2,3,4,5,6,7,8,9,10,11].

Tsuji et al. have systematically studied the stereocomplexation ability of PLA isomers [2,3,4,5,6,7,8,9]. They found that the optimal condition for stereocomplexation from solution is the use of the same molar amount of PLLA and PDLA with similar molecular weights. On the other hand, the stereocomplexation behavior of PLA chains within a copolymer has been studied by Michell et al. [12]. The poly(d-lactide)-*b*-poly(*N*,*N*-dimethylamino-2-ethyl methacrylate) (PDLA-*b*-PDMAEMA) and poly(l-lactide)-*b*-poly(*N*,*N*-dimethylamino-2-ethyl methacrylate) (PLLA-*b*-PDMAEMA) copolymers were able to form stereocomplex structures, however, the crystallization temperature was lower than that of the stereocomplex formed by the homopolymers, this indicated that the crystallization of the stereocomplex is impaired by the amorphous block of PDMAEMA in the copolymers [12].

To enhance the physical properties of PLA, various chemical modifications and blending methods have been employed [13,14,15,16]. PBS was selected in this work due to its biocompatibility and reported application in blending with PLA to improve its mechanical performance [17,18,19,20,21,22,23,24,25,26,27,28]. The copolymerization of PBS and PLA as multi-block copolymers may lead to a material with improved mechanical properties as compared with PLAs [20,26,29]. Additionally, the crystallization process of such multi-block copolymers has not been studied in detail, and the stereocomplexation ability of such copolymers has not been reported in the literature so far. Hence, in this publication, we study the morphology and isothermal crystallization of PLA-*mb*-PBS multi-block copolymer and the stereocomplexes that can be formed by mixtures of copolymers and by PLA-*mb*-PBS copolymer/PLA homopolymer blends.

## 2. Materials and Methods

### 2.1. Materials

The molecular weights and polydispersities of the employed materials are shown in Table 1, the nomenclature used is as follows: (L or D)L*_xx_^zz^*-*mb*-BS*_yy_^zz^*, where *xx* and *yy* represent the content (in weight percent) of the PLA and PBS blocks, respectively. The superscript *zz* represents the number-average molecular weight in kg/mol. Some of us have reported the complete synthesis procedure of all studied block copolymers and homopolymers previously [21].

### 2.2. Stereocomplex Preparation

PLLA and PDLA homopolymers were dissolved in dichloromethane, separately, at a fixed concentration of 1 g/dL. Then PLLA and PDLA solutions with similar molecular weights (see Table 2) were mixed together under vigorous stirring for 3 h, the molar ratio of PLLA/PDLA in all the blends was fixed at 1:1. Finally, the mixed solutions were casted onto Petri dishes and the solvent was evaporated at room temperature for 24 h. The product was dried under vacuum at 25 °C for 72 h.

The same solution method was employed to prepare PLA-*mb*-PBS copolymers and copolymer/homopolymer blends. Table 2 summarizes all stereocomplex samples prepared with homopolymers, copolymers, and their blends.

### 2.3. Polarized Light Optical Microscopy (PLOM)

The superstructural morphology was observed employing a ZEISS Mc 80 polarized light optical microscope (Carl Zeiss Microscopy, Jena, Germany). The homopolymers and copolymers were melted at 180 °C and the stereocomplexes at 230 °C in order to erase the thermal history. Next, the temperature was quickly lowered until the desired isothermal crystallization temperature was reached. The morphology was recorded with a digital camera and the temperature was controlled using a Linkam TP 91 hot plate (Linkam Scientific Instruments, Tadworth, UK).

### 2.4. Differential Scanning Calorimetry (DSC)

Homopolymer/homopolymer equimolar blends were characterized by differential scanning calorimetry with a Perkin-Elmer DSC-7. For copolymer/copolymer and homopolymer/copolymer blends, DSC characterization was performed with a Perkin-Elmer DSC Diamond^TM^ (Perkin Elmer, Waltham, MA, USA). The samples were studied under inert atmosphere employing ultra-high purity nitrogen and the instruments were calibrated with indium and tin standards. The non-isothermal crystallization studies were performed at 20 °C/min. For isothermal crystallization, the samples were quenched from the melt at 60 °C/min to each isothermal crystallization temperature investigated and then measurements as a function of time were performed until the crystallization was complete, following the procedure reported by Lorenzo et al. [30].

### 2.5. Infrared Spectroscopy (FTIR)

For FTIR experiments of PLLA/PDLA stereocomplexes, the samples were prepared by compression moulding of discs of KBr/sample blends. The discs were placed in the sample compartment of a Nicolet Magna 760 spectrometer (Thermo Fisher Scientific, Waltham, MA, USA) at room temperature. Thirty-two scans were co-added in order to achieve an acceptable signal-to-noise ratio.

### 2.6. Carbon-13 Nuclear Magnetic Resonance Spectroscopy (^13^C-NMR)

Solid-state NMR spectra were obtained using a Bruker Avance TM 400 WB spectrometer (Bruker, Billerica, MA, USA) operating at 400 MHz for ^13^C using cross-polarization (CP), magic-angle spinning (MAS), and high-power ^1^H decoupling. The contact time was set to 3 ms, and recycle time between subsequent acquisitions was set to 3 s. A total of 2092 data points were acquired using a spectral width of 30 kHz. A total of 2000 and 1536 transients were averaged for each spectrum. MAS speed was between 5 kHz and 7.5 kHz.

## 3. Results and Discussion

### 3.1. Homopolymers and Copolymers

#### 3.1.1. Non-Isothermal Crystallization

The Fox equation (see Equation (1)) was applied to estimate the *T_g_* values for a miscible blend or copolymer, depending on the composition and on the *T_g_* values of the original homopolymers [31]. Table 3 shows the values estimated using the Fox equation and the experimental ones.

(1) $$\frac{1}{T_{g}}=\frac{X_{A}}{T_{gA}}+\frac{X_{B}}{T_{gB}}$$

A single glass transition temperature was found for all copolymers employed here. The *T_g_* values experimentally determined are between those of PBS and PLA and they decrease with the increase of PBS content within the copolymer. The experimental values differ from those predicted by the Fox equation; however, their decrease with the increase in PBS content suggests that the copolymers form a single phase in the melt (i.e., homogeneous melt), where both PBS and PLA chains are mixed.

**Figure 1:**
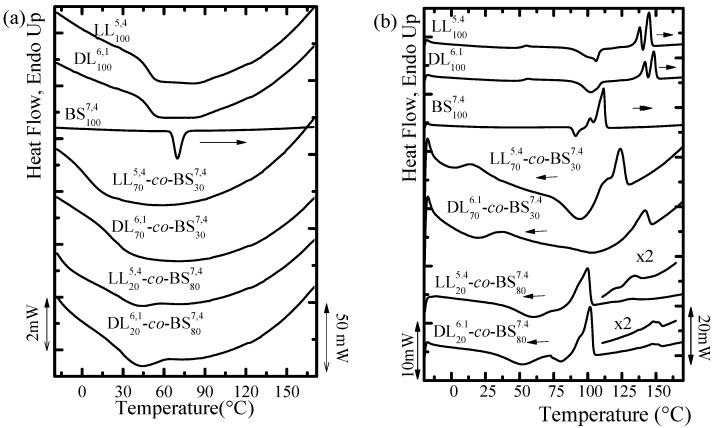
DSC scans at 20 °C/min for the homopolymers and copolymers: (**a**) cooling; and (**b**) second heating.

The influence of the copolymerization process on the crystallization and melting of both PBS and PLA blocks is shown in Figure 1. The crystallization process can be observed in Figure 1a for the PBS homopolymer and for the PBS block within the copolymers with 80% of PBS (LL_20_^5.4^-*mb*-BS_80_^7.4^ and DL_20_^6.1^-*mb*-BS_80_^7.4^). In the case of those copolymers with 30% PBS, the crystallization of the PBS blocks cannot be detected during cooling from the melt.

During the cooling scans from the melt, it is not possible to detect any crystallization exotherm corresponding to PLA homopolymers or PLA phases within the copolymers. However, during the subsequent heating scans, it is possible to observe cold crystallization exotherms that can be attributed to PLA homopolymers or to the PLA blocks within the copolymers. It is well known that PLA homopolymers have a slow crystallization kinetics when cooled from the melt, but during cooling and vitrification their nucleation density can be greatly enhanced. Therefore, when heated from the glassy state they can undergo cold-crystallization [32,33,34,35].

During the second heating scan, PLLA and PDLA homopolymers melt at around 140 °C (see Table 4) while PBS melts at 110 °C. This large difference in melting temperatures is useful to distinguish between the melting of PBS block crystals from those of PLA block crystals within the copolymers. The multi-block copolymers with the lowest PBS content (LL_70_^5.4^-*co*-BS_30_^7.4^ and DL_70_^6.1^-*co*-BS_30_^7.4^) show only one melting peak that corresponds to the fusion of the PLA block crystals. Therefore, in these copolymers the PBS blocks are unable to crystallize during cooling or during the subsequent heating and remain amorphous.

On the other hand, the copolymers with 80% PBS are double crystalline, as they exhibit two clear melting endotherms that can be ascribed to the sequential melting of PBS block crystals (at around 100 °C) and PLA block crystals (at temperatures between 125–150 °C).

The melting process in both PLLA and PDLA is characterized by two endothermic peaks. PLAs have a tendency to reorganize during heating and this is probably the origin of such double endotherms. Another possibility, would be the presence of two polymorphs, i.e., the α′ and α phases, but such a possibility would have to be assessed by wide angle X-ray diffraction. The difference between the melting temperatures of PDLA and PLLA homopolymers may originate from their small difference in molecular weight, as in this molecular weight range (i.e., very low molecular weights), the melting point is a strong function of the average molecular weight [36].

In the copolymers case, the incorporation of PBS segments covalently bonded to PLA chains decreases the melting point values corresponding to the PLA blocks; this could be attributed to a dilution effect, since these di-block copolymers are miscible in the melt [32,33,34]. Therefore, the PBS blocks act like a macromolecular solvent surrounding the PLA crystals in the melt. In addition, the covalent link between the PLA and PBS will affect the crystallization process of both blocks and, consequently, their corresponding *T_c_* and *T_m_* values [37].

The PBS endotherm has a complex behavior as well, as it is possible to observe a cold crystallization peak before the double melting endotherm. In this case, cold crystallization is followed by the melting of the crystals formed during previous cooling (first endothermic peak) while the second peak is a consequence of the melting of the reorganized crystals during the scan.

#### 3.1.2. Polarized Light Optical Microscope: Morphology and Superstructural Growth Kinetics

**Figure 2:**
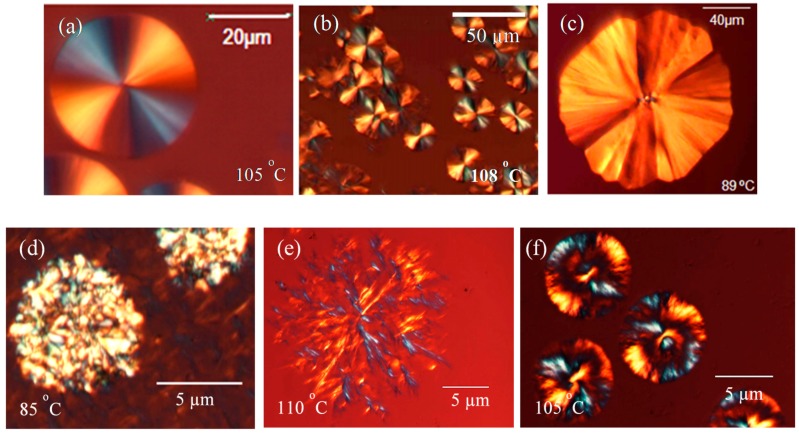
PLOM micrographs for (**a**) DL_100_^6.1^; (**b**) LL_100_^5.4^; (**c**) BS_100_^7.4^; (**d**) LL_70_^5.4^-*mb*-BS_30_^7.4^; (**e**) LL_20_^5.4^-*mb*-BS_80_^7.4^; and (**f**) DL_20_^6.1^-*mb*-BS_80_^7.4^, during isothermal crystallization at the indicated *T_c_*.

The samples were isothermally crystallized from the melt on a hot plate and were observed employing PLOM. The superstructural morphology of the homopolymers and copolymers are shown in Figure 2.

Both PLA homopolymers (DL_100_^6.1^ and LL_100_^5.4^) display negative spherulites at low crystallization temperatures (with no banding) which are similar to those shown in Figure 2a,b. At higher temperatures, the spherulites show banding extinction patterns.

In the case of neat PBS, the superstructure was spherulite-like at lower crystallization temperatures, although the spherulites did not appear to have a perfectly circular cross-section. When the crystallization temperature was increased, hedrites were formed, instead of spherulites, as revealed by the example shown in Figure 2c.

**Figure 3:**
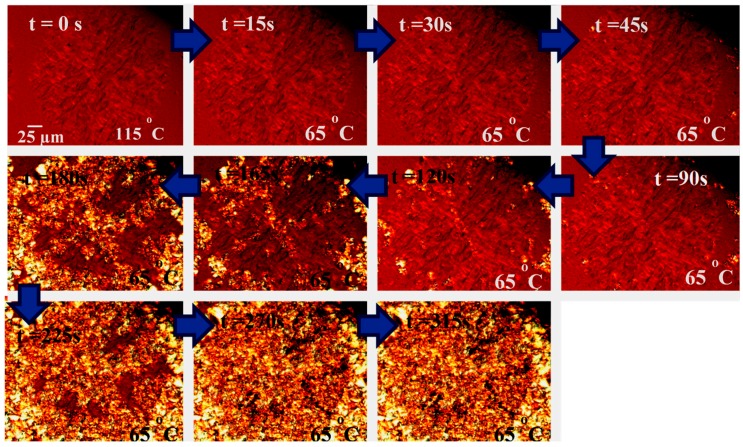
PBS crystallization at 65 °C from a PLLA spherulite (*T_c_* = 115 °C) within a LL_20_^5.4^-*mb*-BS_80_^7.4^ copolymer.

For the copolymers with high PLA content, the superstructures observed at high *T_c_* values (higher than the crystallization temperature of the PBS block) were distorted spherulites with ill-defined banding, as shown in Figure 2d. The most interesting fact is the crystallization of the PBS component within the interlamellar regions of the PLA template (formed at higher temperatures) when the crystallization temperature is reduced to values where PBS is able to crystallize (see Figure 3). In Figure 2d it is possible to observe the birefringence due to PBS interspherulitic lamellae (brighter regions of the sample) inside the PLA component template superstructure. In the case of the copolymers with a lower content of PLA, the superstructures observed are more similar to dendrites (see Figure 2e).

Figure 3 shows a sequence of polarized light micrographs taken at different times and temperatures for copolymer LL_20_^5.4^-*mb*-BS_80_^7.4^. The sample was first isothermally crystallized at 115 °C, a temperature at which the PBS blocks are molten. A super-structural template is formed by the part of the PLA component that can crystallize, as can be observed in the first micrograph (top left-hand corner of Figure 3). As this copolymer sample contains only 30% PLA, the amount of crystalline superstructural phase observed in the micrograph could be at most 15% of the total sample by weight. Then, the sample was quenched to 65 °C, since at this temperature the isothermal crystallization of the PBS component can be followed as a function of time.

Figure 3 shows how the PBS component is nucleated by the PLA block crystals, forming PBS crystalline aggregates inside and on top of the PLA previously formed a loose template superstructure. As time increases, the entire PLA template is filled with PBS crystals. PBS and PLA blocks are melt mixed, but will phase segregate as PLA and PBS crystallize. Their amorphous regions still remain mixed, forming a single phase characterized by a single *T_g_* value. As the amount of initial PLA crystals is in minority (i.e., equal or less than 15% of the sample), confinement of the PBS blocks during crystallization is not expected, as PBS blocks constitute the more abundant component in the multi-block component.

We tried to determine the growth rate of the different crystalline structures but their growth was very difficult to follow, especially when the morphology was not spherulitic. Therefore, we decided to determine, instead, the overall crystallization kinetics of selected samples by DSC.

#### 3.1.3. Overall Isothermal Crystallization Kinetics

**Figure 4:**
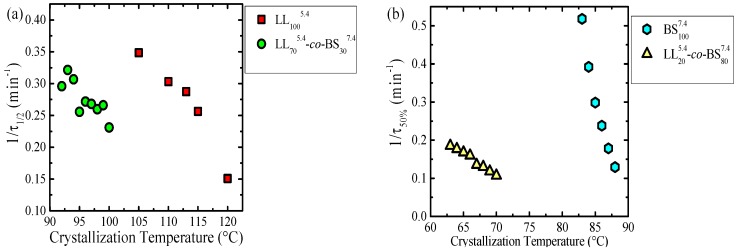
Overall crystallization rate (1/τ_50% exp_) versus crystallization temperature (*T_c_*) for (**a**) PLLA homopolymer and PLLA blocks within the LL_70_^5.4^-*mb*-BS_30_^7.4^ copolymer; and (**b**) PBS homopolymer and PBS blocks within LL_20_^5.4^-*co*-BS_80_^7.4^ copolymer.

The study of the isothermal crystallization from the melt was performed for the samples that contained PLLA, instead of PDLA, as the measurements were facilitated by their generally faster crystallization from the melt. The overall crystallization rate, expressed as the inverse of the peak-crystallization time (1/τ_50%_), as a function of the isothermal crystallization temperature, is shown in Figure 4.

The overall crystallization kinetics comprises both primary nucleation and growth. The balance between these two processes will determine how the overall crystallization rate will be affected by the crystallization temperature and other factors, like molecular weight or composition. In Figure 4, it is possible to observe that the crystallization of the PLLA and PBS blocks need a higher undercooling than the respective homopolymers. This is a consequence of the miscibility between the blocks which changes the relative supercooling, as the equilibrium melting temperature must be reduced in the copolymers, as compared to the homopolymers. For the copolymer with a low content of PLLA it was impossible to measure the isothermal crystallization, as in this case the amount of crystallizable material was too small, and the calorimeter could not detect any signal in the isothermal mode.

Before the PBS isothermal crystallization, the PLLA block within the LL_20_-*mb*-BS_80_ copolymer was isothermally crystallized until saturation. Then the sample was quickly cooled (at 60 °C/min) to the PBS crystallization temperatures. It is worth pointing out that only the copolymer sample with 80% PBS content was examined, as in the samples with minor amounts of PBS, this component was unable to crystallize. The presence of rigid PLA semi-crystalline blocks retarded the isothermal crystallization kinetics of the PBS blocks within the LL_20_^5.4^-*co*-BS_80_^7.4^ copolymer, in comparison to the analog neat PBS.

The experimental data obtained during the overall isothermal crystallization experiments were analyzed using the Avrami equation, which can be expressed as follows:

(2) $$1-V_{c}\left(t-t_{0}\right)=e^{\left(-k{\left(t-t_{0}\right)}^{n}\right)}$$

where *t* is the experimental time, *t*_0_ is the induction time, *V_c_* is the relative volumetric transformed fraction, *n* is the Avrami index, and *k* is the overall crystallization rate constant [30,38].

The results are shown in Table S1 for PLLA and Table S2 for PBS homopolymers and corresponding blocks. The Avrami indices obtained for the PLLA homopolymer can be approximated to values between 3 and 4. These values are in agreement with instantaneous or sporadic three-dimensional spherulitic structures, as observed by PLOM (see above). For the PLLA block within LL_70_^5.4^-*mb*-BS_30_^7.4^, the most frequently obtained values were around 3 (with a few exceptions), indicating instantaneously nucleated three-dimensional superstructures in correspondence with those observed in Figure 2.

According to PLOM observation, the low molecular weight PBS employed here displayed hedrites, which are 2D lamellar aggregates that characterize the transition from axialites to spherulites. The Avrami index obtained for neat PBS was between 2.5 and 2.7, suggesting 2D aggregates with a sporadic nucleation, in agreement with PLOM observations. In the case of the PBS blocks crystallized within LL_20_^5.4^-*co*-BS_80_^7.4^ copolymer, similar n values (between 3.2 and 2.8) were found.

## 4. Stereocomplexes

### 4.1. Non-Isothermal Crystallization

**Figure 5:**
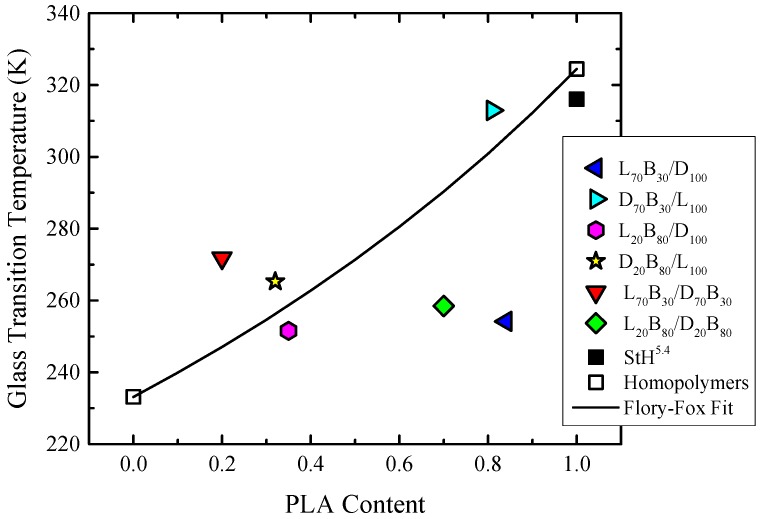
Glass transition temperature (*T_g_*) versus PLA content (XPLA) for different homopolymers, copolymers and copolymer/homopolymer blends. The solid line represents the fitting to the Fox equation.

As previously observed for the copolymers (see Table 3), all the blends exhibited a single glass transition, denoting that the samples have a single phase in the melt and in the amorphous state. The *T_g_* results are summarized in Figure 5. In the copolymer/copolymer and copolymer/homopolymer blends, *T_g_* decreases as the PBS content increases. This behavior corresponds to the plasticizating ability of the PBS segments. However, for the blends with DL homopolymer the glass transition is lower than the samples with LL homopolymer (with the same composition) (see Figure 5 and Table 5).

**Figure 6:**
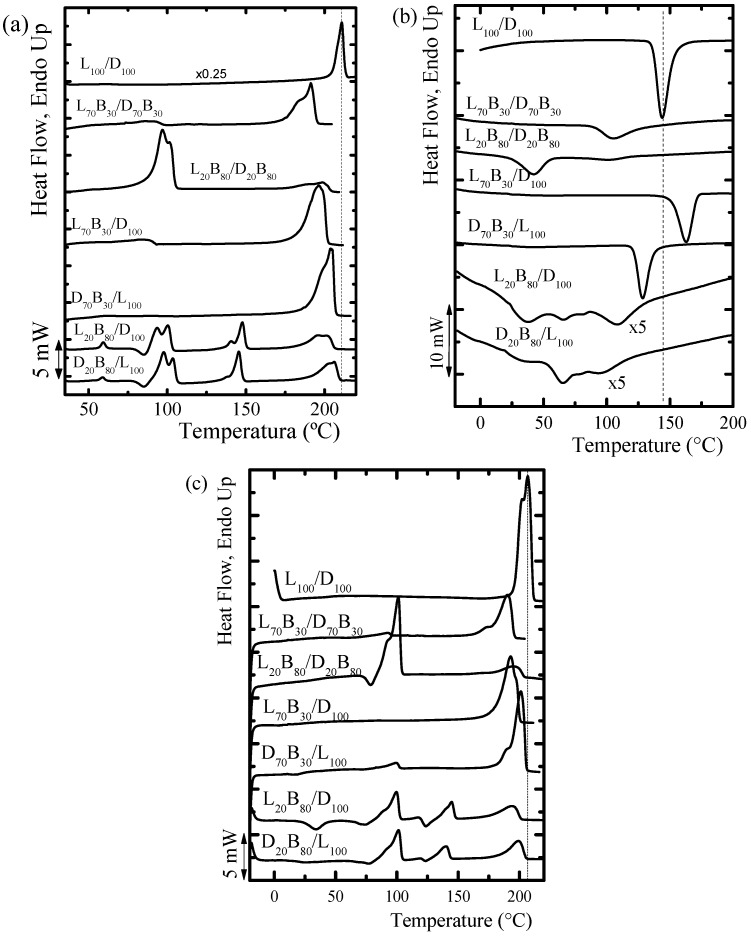
Non-isothermal DSC (**a**) first heating, (**b**) cooling, and (**c**) second heating scans at 20 °C/min for the indicated stereocomplexes. The dashed line represents the peak crystallization and melting temperature for the L_100_/D_100_ stereocomplex.

The stereocomplex crystals were obtained by precipitation from solutions of equimolecular mixtures. The first DSC scans of the samples show the characteristics of the crystals formed from solution. Figure 6a shows DSC first heating scans for all the blends prepared. All the samples show melting peaks at around 190–210 °C, these *T_m_* values are higher than those of PLA homocrystals (crystals formed by homopolymers), indicating the presence of stereocrystals. Stereocomplexation of PLLA and PDLA chains is well known. PLLA and PDLA display a strong interaction with each other and can share a new crystal lattice. The stereocomplex unit cell is formed by segments of PLLA and PDLA packed in parallel, and the complex forms a 3_1_ helical conformation, which is derived from the 10_3_ helical structure characteristic of PLA homopolymers α phase. The temperature difference obtained for *T_m_* stereocomplex crystals and *T_m_* PLA homopolymers crystals (of approximately 50 °C) implies a significant change between the two crystal structures [4,5,6,9]. As expected, the L_100_/D_100_ equimolar blend only forms stereocomplexes when crystallized from solution (i.e., no homocrystals are formed) and its *T_m_* value is the highest shown in Figure 6a.

Figure 6a also shows the first heating scan of the copolymer/copolymer and copolymer/homopolymer blends, and it is possible to observe multiple endothermic peaks in almost all the samples. The endotherms at around 100 °C correspond to the melting of PBS crystals and they are present in most samples, with the exception of the sample D_70_B_30_/L_100_ where the PBS component does not crystallize.

The samples L_20_B_80_/D_100_ and D_20_B_80_/L_100_ in Figure 6a are the only ones that show endothermic peaks associated with the melting of the PLA homocrystals (at temperatures around 140 °C). The presence of homocrystals in these samples could be due to their difficulty in forming stereocomplexes. For example, in the L_20_B_80_/D_100_ blend, PDLA has a higher probability of finding another PDLA segment to form homocrystals than the possibility of finding a PLLA chain to form a stereocrystal. These two samples are also the only ones where the PBS blocks exhibit cold crystallization exotherms just before melting in double-peaked endotherms, which are due to reorganization during the scan.

The highest melting temperature (*T_m_*) observed in Figure 6a corresponds to homopolymer/homopolymer blends. Since it is higher than those of stereocomplexes formed by copolymer blends, this is an indication that the presence of PBS in the multi-block copolymers disturbs the formation of the stereocrystals. The latent enthalpy of fusion of the PLLA/PDLA homopolymer/homopolymer stereocomplex is the highest of all stereocomplexes reported in Table 5. The enthalpy of fusion for the stereocomplexes prepared from copolymer/copolymer blends decreases as the content of PLA decreases, as stereocomplexation becomes more difficult. A similar effect is observed for copolymer/homopolymer blends in Table 5.

Figure 6b shows the DSC cooling scans from the melt, while Figure 6c shows the subsequent heating scans (i.e., second heating scans). Upon cooling from the melt, it is expected that the L_100_/D_100_ mixture exhibits the highest crystallization temperature as stereocomplexation would be easier for this sample in comparison with all others. This is, in fact, observed, as almost all samples crystallize at lower temperatures, with the exception of the L_70_B_30_/D_100_ sample. In the case of samples with small amounts of PLA, the crystallization exotherms are broad and multimodal, as at least three different types of crystals are formed: PLA stereocomplex crystals, PLA homocrystals and, finally, PBS crystals.

Figure 6c shows the heating scan after melt crystallization. When Figure 6c is compared to Figure 6a (for solution crystallized samples), it can be seen that the behavior in both cases is very similar. The stereocomplexes in the copolymer/copolymer and copolymer/homopolymer blends were formed from the melt without any problems. The only exception observed was that of sample D_70_B_30_/L_100_, which was able to form a very small amount of PLLA homocrystals when crystallized from the melt, and none when crystallized from solution.

**Figure 7:**
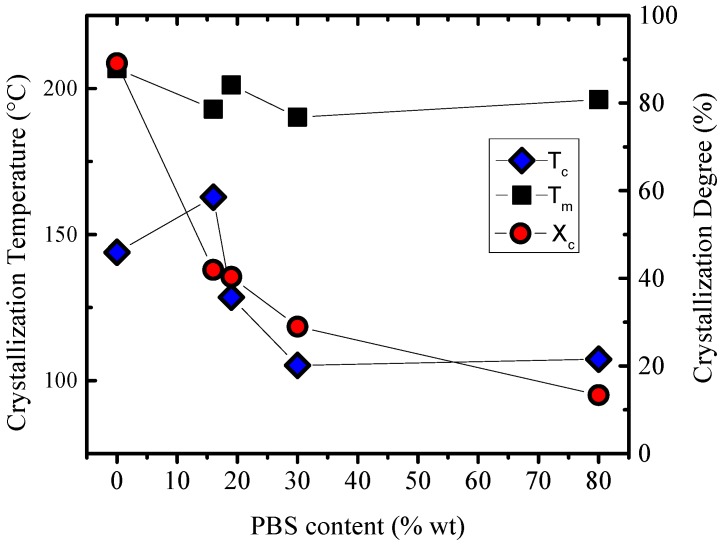
Crystallization degree, crystallization, and melting temperature versus the PBS content for the copolymer/copolymer and copolymer/homopolymer stereocomplexes.

The PBS blocks act as a plasticizer for the PLA blocks in the copolymers and in their blends, as was demonstrated for studies in PBS-*b*-PLA-*b*-PBS triblock copolymer [20] and as was discussed in the previous sections. Figure 7 was drawn in order to understand the role of PBS content in the crystallization of copolymer/copolymer and copolymer/homopolymer stereocomplexes. In general, increasing the amount of PBS in the blends depresses the crystallization temperature, as expected, since PBS causes a dilution effect on PLA stereocomplexes.

The degree of crystallinity was calculated from Δ*H_m_* values (melting enthalpies determined from the second heating scans) for stereocrystals and homocrystals, employing the following values for the enthalpies of fusion of 100% crystalline materials: Δ*H_m_*°: PBS (110.3 J/g) (66), PLA (93.6 J/g) (67), and PLA stereocomplex (142 J/g) (68). In general, crystallinity degree of PLA stereocomplexes decreases with increasing PBS content (see Figure 7). Consequently, fewer PLA stereocomplex crystals were formed for the L_20_B_80_/D_20_B_80_ blend and a higher proportion of PBS crystals. However, PBS does not prevent PLA stereocomplex formation even if PBS is the major component in this blend. The amount of stereocomplexes decreases when PLA content decreases, as too much PBS can generate a “macromolecular solvent” matrix where dispersed PLA molecules will have large mobility, but are highly diluted.

### 4.2. Polarized Light Optical Microscope: Stereocomplex Superstructural Morphology

**Figure 8:**
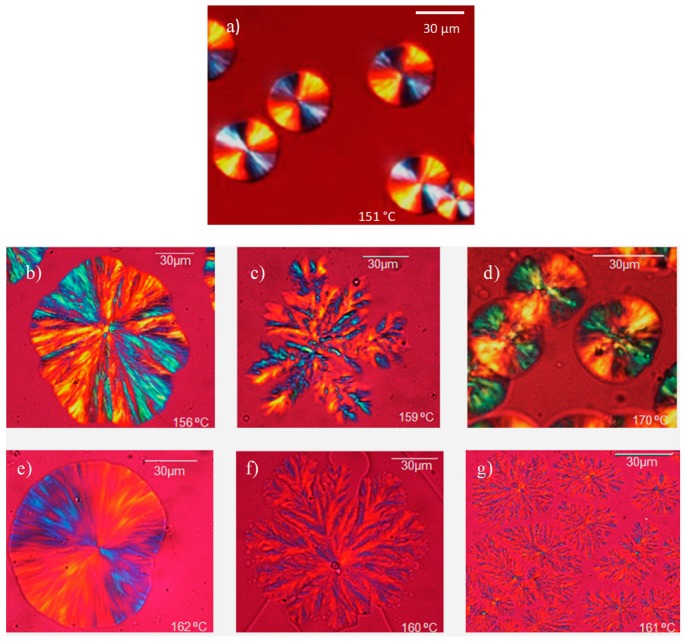
PLOM micrographs for (**a**) L_100_/D_100_; (**b**) L_70_B_30_/D_70_B_30_; (**c**) L_20_B_80_/D_20_B_80_; (**d**) L_70_B_30_/D_100_; (**e**) D_70_B_30_/L_100_; (**f**) L_20_B_80_/D_100_; and (**g**) D_20_B_80_/L_100_, during isothermal crystallization at the indicated *T_c_*.

Figure 8 shows the stereocomplex superstructures observed in a polarized light optical microscope during isothermal crystallization at high *T_c_* values, where only stereocomplex can crystallize (i.e., the *T_c_* values employed are higher than the melting points of PLA homocrystals and PBS crystals).

For PLA homopolymer stereocomplexes the structures observed correspond to negative spherulites with Maltese cross extinction patterns. On the other hand, the copolymer/copolymer and copolymer/homopolymer blends exhibit a variety of superstructural morphologies. The stereocomplexes formed by LL_70_^5.4^-*mb*-BS_30_^7.4^/DL_70_^6.1^-*mb*-BS_30_^7.4^, LL_70_^5.4^-*mb*-BS_30_^7.4^/DL_100_^6.1^, and DL_70_^6.1^-*mb*-BS_30_^7.4^ also display negative spherulites; however, the other two stereocomplexes (i.e., L_20_B_80_/D_100_ and D_20_B_80_/L_100_) exhibit a dendritic structure in view of their low PLA content [39]. The obtained morphologies are a function of composition. When the majority of the blends are constituted by PLA isomers, the morphology corresponds to 3D spherulites, and when the PLA content is small, then 2D aggregates (or axialites) developed. The results parallel those obtained for PLA homocrystals above (see Figure 2).

The morphology for the blends with high amounts of PBS segments (L_20_B_80_/D_20_B_80_, L_20_B_80_/D_100_ and D_20_B_80_/L_100_) was always dendritic, as the concentration of PLA chains is rather low. Figure 8c,f,g represent mixtures with a higher PBS proportion. In this case, dendrites with secondary branches, called dizzi dendrites, can be observed. These dizzi dendrites are polycrystalline structures formed by sequential deformation of the tips of dendrites. Their formation mechanism has not been identified [39].

### 4.3. Overall Isothermal Crystallization Kinetics of Stereocomplexes

**Figure 9:**
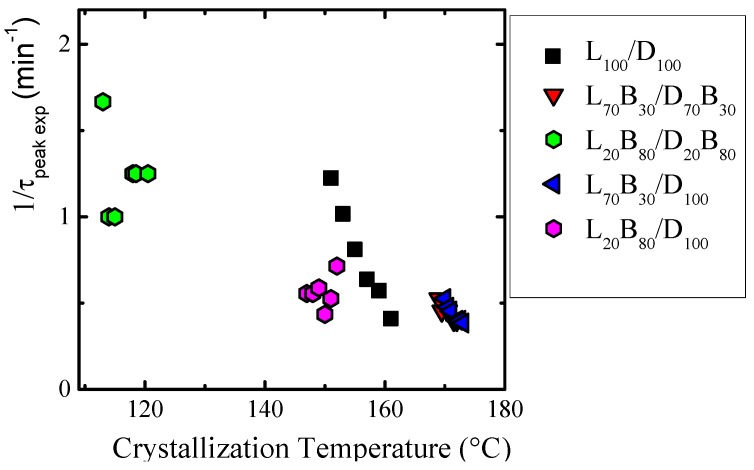
Overall crystallization rate (1/τ_peak exp_) versus crystallization temperature (*T_c_*) for PLA stereocomplexes.

The overall crystallization rate (estimated as the inverse of the half-peak crystallization time) versus crystallization temperature for selected stereocomplexes is plotted in Figure 9. The results obtained are a function of PLA content in the samples.

In the case of copolymer/copolymer or copolymer/homopolymer blends, where the copolymer contains 70% PLLA, the stereocomplexes were formed at lower supercoolings than L_100_/D_100_ stereocomplexes (see Figure 9). This is a remarkable result, especially when a comparison is made with the plasticizing effect of the PBS block chains have on PLLA homocrystals, in the case of neat copolymers (see Figure 4). It would seem that for stereocomplex formation, molten PBS segments plasticize the PLA chains, helping them find the necessary PDLA/PLLA pairs for stereocomplexation. As a result, the stereocomplex formation occurs faster when PBS chains are present in the copolymer/homopolymer blends. The situation changes when the number of PLA chains in the copolymer is reduced. In that case, it seems that too much PBS causes a dilution effect on PLLA and PDLA chains, causing difficulties for the formation of stereocomplexes and, therefore, a higher supercooling is needed for stereocomplexation, as compared to the simpler L_100_/D_100_ case.

According to the results presented in Figure 6 and Figure 9, the effect of the PBS blocks on the formation of PLA stereocomplexation can be ascertained. According to their melting points and enthalpies after non-isothermal crystallization, the PBS segments induce a lower amount of stereocomplex formation as compared to neat L_100_/D_100_ stereocomplexes. It seems that PBS perturbs the formation of stereocomplexes and this perturbation increases with the amount of PBS in the samples. However, when relatively low amounts of PBS in the copolymer blends is present, the rate of stereocomplex formation is enhanced (if not the crystallinity degree achieved). This effect is lost when higher amounts of PBS are present.

### 4.4. Infrared Spectroscopy for PLA Stereocomplexes (FTIR)

According to the literature, PLA stereocomplex formation involves the formation of double helices. The molecules adopt a 3_1_ helical conformation known as the β-form and a segment of PLLA molecule is paired with a segment of PDLA molecule, resulting in a triclinic unit cell [40]. In a permanently polar bond, the partial charge of this dipole will attract the partial opposite charge in the other molecule. This is a result of the asymmetric distribution of regions with positive and negative charges. This distribution can induce dipoles in nonpolar molecules. A positively-charged molecule is capable of generating an attractive force with the negative end of an adjacent molecule, which justifies the formation of C-H···O bonds presented by PLA stereocomplexes.

**Figure 10:**
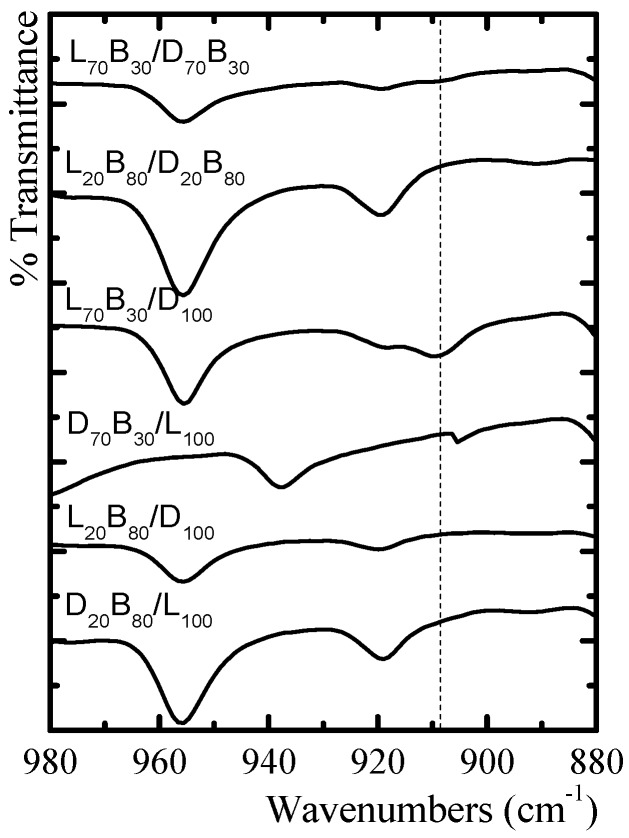
Infrared spectrum of the PLA stereocomplexes. Sensitive bands to the formation of PLA stereocomplexes. The dashed line indicates the position of the 909 cm^−1^ band.

The IR band at 909 cm^−1^ is characteristic of the 3_1_ helical conformation of stereocomplex PLA crystals. The presence of this band depends on the crystallinity of the polymer and the helix type [41]. The intensity of the stereocomplex band is higher in the samples with lower molecular weight (see Figure 10) and higher PLA content, this is a consequence of a larger content of PLA stereocomplex in these samples, as demonstrated by DSC. The FTIR results presented in Figure 10 are able to prove the existence of the PLA stereocrystals, however, it is necessary that a high amount of stereocrystals are present to produce a signal. For some of the samples in Figure 10, the 909 cm^−1^ signal is reduced because of the presence of PBS chain segments and PLA homocrystals.

### 4.5. Carbon-13 Nuclear Magnetic Resonance Spectroscopy (^13^C-NMR) for PLA Stereocomplexes Prepared from Copolymer-Homopolymer Blends

**Figure 11:**
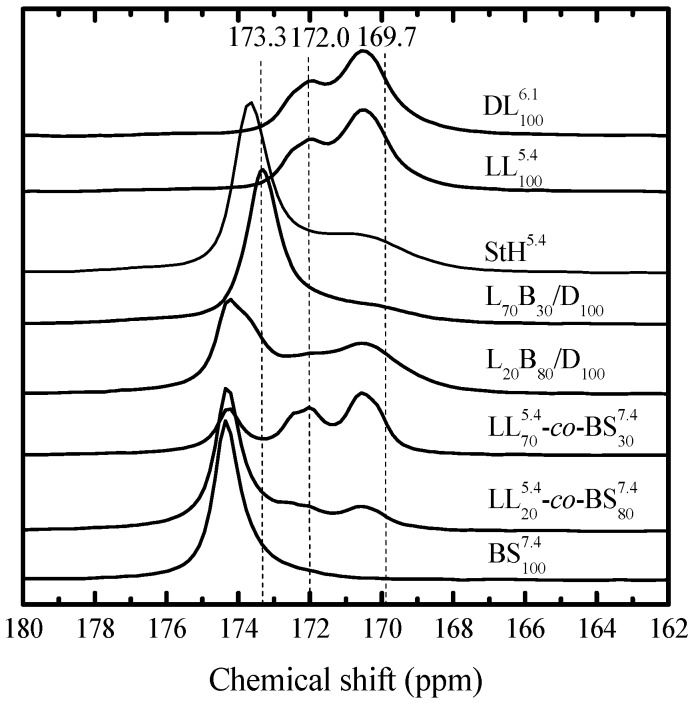
Proton-decoupled ^13^C spectrum of homopolymers, copolymers, and mixtures thereof to form PLA stereocomplexes (400 MHz).

In order to further characterize the presence of stereocrystals, ^13^C-NMR experiments were performed in the solid state. The carbon-13 nuclear magnetic resonance spectra of selected samples are shown in Figure 11. The spectra corresponding to the homopolymers PDLA and PLLA present two broad peaks fixed at 170.4 ppm assigned to the non-crystalline component and 172.0 ppm assigned to the crystalline component. The spectrum corresponding to neat PBS presents a resonance peak at 174.5 ppm that corresponds to the carbonyl carbon. The stereocomplex formation in the sample L_100_/D_100_ is ascertained by the presence of a resonance peak at 173.7 ppm which has been previously assigned to the rigid racemic crystalline component overlapped with disordered racemic crystalline component. The spectra corresponding to the copolymers do not show the presence of the peak assigned to the stereocomplex formation. However, in the spectrum corresponding to the sample L_70_B_30_/D_100_ this peak is shifted to 173.3 ppm, whereas for sample L_20_B_80_/D_100_ it appears as a shoulder at 173.7 ppm. These results confirm the formation of the stereocomplex in the case of the copolymer-homopolymer blends.

## 5. Conclusions

The thermal properties and morphologies of homopolymers, copolymers and stereocomplexes of PLA-*mb*-PBS multi-block copolymers were investigated. The PLA and PBS multi-blocks within the PLA-*mb*-PBS copolymers are miscible according to the observation of a single *T_g_* value. The PBS has a dilution effect on PLA blocks, increasing the PLA chain mobility, thus lowering the crystallization and melting temperature and increasing the undercooling during isothermal crystallization. The Avrami theory was able to fit properly the data of all analyzed homopolymers and copolymers, and the Avrami indices obtained are in line with PLOM observations. Both homopolymers and copolymers with a higher PLA content exhibit negative spherulites, however, the copolymers with a lower PLA content had a dendritic morphology.

The PLA chains stereocomplexes were formed from solution and from the melt and exhibited melting and crystallization temperatures higher than the original homocrystals, as expected. The decrease of both the melting temperature and the crystallinity degree with PBS content, indicates that the PBS blocks perturb the PLA blocks stereocomplexation. However, the PBS blocks could retard or increase the crystallization kinetics, depending on the PBS amount in the blend. Lower contents of PBS induce a faster crystallization rate of the stereocomplexes, however, a higher content retards it.

The superstructural morphology of the homopolymer/homopolymer stereocomplexes is composed of three-dimensional spherulites, in a similar manner, the copolymer/copolymer and copolymer/homopolymer with the lowest PBS content also displayed a spherulitic texture. However, the superstructures change when the PBS content increases, and in the samples with the largest PBS content the morphology of the stereocrystals was dentritic.

The formation of stereocrystals was verified employing solid state ^13^C-NMR and FTIR. The characteristic infrared absorption band at 909 cm^−1^, associated with stereocomplex formation, was observed only in copolymer-homopolymer blends with a higher PLA content.

## Figures and Tables

**Table 1:** Molecular characteristics of the PLA and PBS homopolymers and copolymers.

Sample	*M_n_* (g/mol)	*M_w_*/*M_n_* ^c^
LL_100_^5.4^	5400 ^a^	1.14
DL_100_^6.1^	6050 ^a^	1.19
BS_100_^7.4^	7400 ^b^	1.77
LL_70_^5.4^-*mb*-BS_30_^7.4^	14,800	1.91
DL_70_^6.1^-*mb*-BS_30_^7.4^	12,000	1.90
LL_20_^5.4^-*mb*-BS_80_^7.4^	7000	1.87
DL_20_^6.1^-*mb*-BS_80_^7.4^	6700	2.86

^a^ The calculated number average molecular weights using reported Mark-Houwink parameters; ^b^ the apparent number average molecular weights determined by size exclusion chromatography in THF at 30 °C with reference to polystyrene standards; ^c^ the molecular weight dispersities.

**Table 2:** Identification of PLA stereocomplexes from PLA homopolymers and copolymers blends.

Sample	Components		Global Composition	
	PLLA	PDLA	%PLA	%PBS
L_100_/D_100_	LL_100_^5.4^	DL_100_^6.1^	100	0
L_70_B_30_/D_70_B_30_	LL_70_^5.4^-*mb*-BS_30_^7.4^	DL_70_^6.1^-*mb*-BS_30_^7.4^	70	30
L_20_B_80_/D_20_B_80_	LL_20_^5.4^-*mb*-BS_80_^7.4^	DL_20_^6.1^-*mb*-BS_80_^7.4^	20	80
L_70_B_30_/D_100_	LL_70_^5.4^-*mb*-BS_30_^7.4^	DL_100_^6.1^	84	16
D_70_B_30_/L_100_	LL_100_^5.4^	DL_70_^6.1^-*mb*-BS_30_^7.4^	81	19
L_20_B_80_/D_100_	LL_20_^5.4^-*co*-BS_80_^7.4^	DL_100_^6.1^	35	65
D_20_B_80_/L_100_	LL_100_^5.4^	DL_20_^6.1^-*co*-BS_80_^7.4^	32	68

**Table 3:** Values of *T_g_* estimated employing the Fox equation (*T_g FOX_*) and the experimental values (*T_g exp_*) from the second heating scan at 20 °C/min for the indicated samples.

Sample	*T_g FOX_*	*T_g exp_*
	(°C)	(°C)
LL_100_^5.4^	-	51.0
DL_100_^6.1^	-	51.8
LL_70_^5.4^-*mb*-BS_30_^7.4^	17	3.8
DL_70_^6.1^-*mb*-BS_30_^7.4^	17	25.4
LL_20_^5.4^-*mb*-BS_80_^7.4^	−26	−16.0
DL_20_^6.1^-*mb*-BS_80_^7.4^	−26	−17.7

**Table 4:** Enthalpy of fusion, glass transition, and melting temperature for the homopolymer and copolymers indicated, during the first heating, cooling, and second heating at 20 °C/min.

Sample	Crystal	Cooling		2nd Heating					*χ_c_*
		*T_c_*	∆*H_c_*	*T_g_*	*T_cc_*	∆*H_cc_*	*T_m_*	∆*H*	
		(°C)	(J/g)	(°C)	(°C)	(J/g)	(°C)	(J/g)	(%)
LL_100_^5.4^	PLA	-	-	51.0	106.1	31.6	144.5	10.2	-
							137.8		
DL_100_^6.1^	PLA	-	-	51.8	102.3	33.4	148.6	11.6	-
							142.2		
BS_100_^7.4^	PBS	69.2	74.4	-	91.0	14.6	111.0	67.6	48
LL_70_^5.4^-*co*-BS_30_^7.4^	PLA	-	-	3.8	93.9	13.6	125.1	11.7	-
DL_70_^6.1^-*co*-BS_30_^7.4^	PLA	-	-	25.4	102.3	13.6	141.5	6.3	-
LL_20_^5.4^-*co*-BS_80_^7.4^	PLA	-	-	−16.0	-	-	131.5	9.5	10
	PBS	40.3	3.0		55.2	26.3	97.9	42.8	39
DL_20_^6.1^-*co*-BS_80_^7.4^	PLA	-	-	−17.7	79.5	19.0	147.2	12.0	-
	PBS	42.7	6.1		50.0	30.1	101.7	47.9	22

**Table 5:** Enthalpy of fusion, glass transition, and melting temperature of PLA stereocomplexes during the first heating, cooling, and second heating at 20 °C/min.

Sample	Crystal	1st Heating		Cooling		2nd Heating					*χ_c_*
		*T_m_*	Δ*H_m_*	*T_c_*	∆*H_c_*	*T_g_*	*T_cc_*	∆*H_cc_*	*T_m_*	∆*H_m_*	
		(°C)	(J/g)	(°C)	(J/g)	(°C)	(°C)	(J/g)	(°C)	(J/g)	(%)
L_100_/D_100_	SC_PLA_	210.9	88.9	143.9	−86.2	42.9	-	-	206.8	89.1	63
L_70_B_30_/D_70_B_30_	PBS	91.9	3.3	-		−14.7	63.7	−6.0	92.6	1.7	-
	SC_PLA_	191.2	37.4	105.2	−52.9		-		190.2	41.1	29
L_20_B_80_/D_20_B_80_	PBS	101.6	56.4	44.1	−45.4	−8.7	78.8	−6.9	101.3	47.1	36
	SC_PLA_	198.6	11.0	107.3	−35.0		-		196.2	19.0	13
L_70_B_30_/D_100_	PBS	87.6	3.1	-		−19.0	-		-		-
	SC_PLA_	196.6	63.0	162.8	−67.1		-		192.9	59.5	42
D_70_B_30_/L_100_	SC_PLA_	203.9	59.5	128.5	−62.1	39.8	-		201.2	57.3	40
L_20_B_80_/D_100_	PBS	100.3	33.8		-	−21.6	74.0	−5.4	99.5	28.0	21
	PLA	147.9	38.9	65.3	−90.3		123.7	−12.0	144.8	24.3	13
	SC_PLA_	202.6	52.9		-		-		193.9	48.0	34
D_20_B_80_/L_100_	PBS	103.6	37.8		-	−7.9	77.0	−18.7	101.2	29.9	10
	PLA	145.5	45.3				123.3	−2.8	140.4	24.1	23
	SC_PLA_	206.3	62.2	108.5	−92.2		-		199.2	54.7	39

## Supplementary Materials

The following are available online at www.mdpi.com/2073-4360/10/1/8/s1, Table S1: Avrami fit parameters for the PLA homopolymers and copolymers, Table S2: Avrami fit parameters for the PBS homopolymer and copolymers.
