# Supplementary material for: Crystallization and Stereocomplexation of PLA-mb-PBS Multi-Block Copolymers
Source: Polymers (Basel). 2017 Dec 22;10(1):8. doi: 10.3390/polym10010008 (PMC6414904; doi:10.3390/polym10010008)
Supplement: Supplementary file 1 [file polymers-10-00008-s001.pdf]

Supplementary Materials

Table S1. Avrami fit parameters for the PLA homopolymers and copolymers.

Sample	T_c (ºC)	n	K (min^{-n})	R^2	$t_{50\% exp}$ (min)	$t_{50\% teo}$ (min)
$LL_{100}^{5.4}$	100	4	0.0327	0.9997	2.13	2.15
	105	3.1	0.025	0.9998	2.87	2.94
	110	2.9	0.0211	0.9999	3.3	3.38
	113	2.9	0.0178	0.9999	3.48	3.53
	115	2.7	0.0165	0.9999	3.9	3.94
	120	3.1	0.0021	0.9999	6.63	6.45
$LL_{70}^{5.4}$-co-$BS_{30}^{7.4}$	92	2.3	0.0531	0.9999	3.38	3.1
	93	2.6	0.0501	0.9995	3.11	2.78
	94	2.6	0.0408	0.9997	3.26	2.93
	95	2	0.0453	0.9981	3.91	3.78
	96	2.5	0.0322	0.9996	3.68	3.45
	97	2.6	0.025	0.9998	3.73	3.5
	98	2.9	0.0179	0.9999	3.85	3.56
	99	2.9	0.0189	0.9998	3.76	3.51
	100	3	0.0109	0.9998	4.33	3.98

Table S2. Avrami fit parameters for the PBS homopolymer and copolymers.

Sample	T_c (ºC)	n	K (min^{-n})	R^2	$t_{50\% exp}$ (min)	$t_{50\% teo}$ (min)
$BS_{100}^{7.4}$	83	2.7	0.1223	0.9999	1.93	2.02
	84	2.7	0.0629	0.9999	2.55	2.44
	85	2.6	0.0332	0.9999	3.35	3.16
	86	2.6	0.0159	0.9999	4.2	4.37
	87	2.6	0.0087	0.9999	5.6	5.29
	88	2.5	0.0047	0.9998	7.73	7.12
$LL_{20}^{5.4}$-co-$BS_{80}^{7.4}$	63	3	0.0044	0.9999	5.4	5.45
	64	3.1	0.0033	0.9999	5.66	5.68
	65	3.1	0.0024	0.9999	5.96	5.97
	66	3.2	0.0018	0.9999	6.26	6.24
	67	2.9	0.0017	0.9999	7.4	7.68
	68	2.9	0.0016	0.9998	7.71	8.05
	69	2.9	0.0011	0.9999	8.48	8.75
	70	2.8	0.0011	0.9999	9.35	9.59
